# Dataset for a qualitative interview study exploring the barriers and facilitators to using and recommending aspirin for cancer prevention

**DOI:** 10.1080/21642850.2025.2463916

**Published:** 2025-02-13

**Authors:** Kelly E. Lloyd, Samuel G. Smith

**Affiliations:** Leeds Institute of Health Sciences, University of Leeds, Leeds, UK

**Keywords:** Cancer prevention, aspirin, Lynch syndrome, qualitative, data sharing

## Abstract

**Introduction:**

Aspirin is increasingly recommended for colorectal cancer prevention for people with Lynch syndrome, who are at higher risk. Before starting aspirin, patients should be reviewed by a healthcare professional for contraindications. We conducted interviews to explore the views of people with Lynch syndrome and healthcare professionals on aspirin for cancer prevention. While open data sharing is increasingly implemented for quantitative research, it is less likely to be adopted for qualitative data. We aimed to create and share a qualitative dataset of the interview transcripts in a restricted access repository.

**Methods:**

We interviewed 15 people with Lynch syndrome and 23 healthcare professionals. Healthcare professionals included general practitioners (GPs), community pharmacists, genetic counsellors, and specialist hospital clinicians (e.g. genetics, gastroenterology). The interview schedule was informed by the Theoretical Domains Framework. Interviews were conducted over video or telephone.

**Results:**

Participants could consent to their anonymised interview transcript being deposited in a restricted repository, with access limited to people using the data for non-commercial research, learning or teaching purposes. Those who did not consent could still participate in the interview. Several transcripts were removed due to identifiability concerns. In total, we deposited 12 transcripts with people with Lynch syndrome, and 8 transcripts with GPs.

**Discussion:**

To safeguard participants’ identities, we fully anonymised the dataset. While this acted to protect participants’ identities, it also potentially reduced its usability due to the removal of rich contextual detail. When sharing qualitative data, it is important to balance confidentiality with data reusability.

## Introduction

Aspirin is increasingly recommended internationally for cancer prevention due to evidence supporting its effectiveness for colorectal cancer risk reduction in the general population (Bosetti et al., 2020; Hurwitz et al., 2021; Rothwell et al., 2010, 2011), and among those at higher risk of cancer (Benamouzig et al., 2003; Burn et al., 2011, 2020; Sandler et al., 2003). One group at higher risk of cancer is people with Lynch syndrome. This is an inherited condition caused by faults in the mismatch repair genes (MLH1, MSH2, MSH6, PMS2, or deletions in EPCAM) (Møller et al., 2017), and increases the risk of developing several cancers including colorectal (Hampel et al., 2005). While Lynch syndrome is not considered to be a rare genetic disorder, with approximately one in 400 people estimated to have the condition, less than 5% are aware (Monahan et al., 2023). To date, approximately 9,000 people have been identified with Lynch syndrome in England (Monahan et al., 2023). In 2020, the National Institute for Health and Care Excellence (NICE) in England and Wales updated their guidance (NG151) to recommend daily aspirin for a minimum of 2 years to prevent colorectal cancer in people with Lynch syndrome (National Institute for Health and Clinical Excellence (NICE), 2020). The guidance was updated after the publication of the CAPP2 trial results, which concluded that participants with Lynch syndrome taking aspirin at 600 mg daily had a significantly reduced risk of colorectal cancer, compared with a placebo control arm (Burn et al., 2011, 2020). While NICE does not recommend a specific dose of aspirin, the guidance states that 150–300 mg are commonly used in clinical practice (National Institute for Health and Clinical Excellence (NICE), 2020).

Despite the benefits of aspirin for cancer prevention, the potential side-effects of the medication can make initiating aspirin a complex decision for patients. Daily aspirin can increase the risk of adverse outcomes, such as gastrointestinal bleeding and peptic ulcers (Lanas & Scheiman, 2007; Roderick et al., 1993). Factors that increase the risk of adverse effects include being older (e.g. ≥ 70 years), having a history of gastrointestinal bleeding, Helicobacter pylori infection, and taking aspirin at higher doses (Lanas & Scheiman, 2007). Due to this, it is important for a person with Lynch syndrome to be assessed by a healthcare professional for aspirin contraindications before starting the medication. Patients with contraindications for taking aspirin may be advised against using the medication. Healthcare professionals may also offer patients aspirin with proton-pump inhibitor (PPI) medication, which can be used to reduce aspirin’s adverse effects (Müller et al., 1997; Simon et al., 1995). Therefore, similar to patients, deciding whether to recommend or prescribe aspirin may also be a complex decision for healthcare professionals.

We recruited people with Lynch syndrome and healthcare professionals to take part in a qualitative interview study. We aimed to explore the barriers and facilitators to implementing the NICE guidance recommending aspirin into clinical practice (Lloyd et al., 2022). We set out to create an open dataset of these interview transcripts to be stored in a data repository. Open data sharing is a key principle advocated by the open science movement, which aims to support research to be more transparent and the results more accessible (Spellman et al., 2017). Open data sharing can increase trust in the study findings, as other researchers are able to access the dataset and reproduce the results (Munafò et al., 2017). Open data sharing also supports data reuse, which is argued to be particularly necessary for research that is publicly funded (Bradley et al., 2020), as closed data that is inaccessible can be considered a waste of public funding.

Despite the benefits of open data, its practice has been less commonly adopted in qualitative research (DuBois et al., 2018). There are important barriers though to sharing qualitative data that need to be considered, including the ethical challenges of sharing sensitive data that could identify participants (Chauvette et al., 2019; Tsai et al., 2016). Furthermore, quantitative terms such as ‘reproducibility’ and ‘verification’ of the data do not translate well to qualitative research, given its subjective nature (Tsai et al., 2016). This raises concerns about the benefits of openly sharing such data. However, it can also be argued that sharing any type of research data is inherently valuable. Research is an expensive and time-consuming process, so openly sharing data provides a cost-effective approach to conducting new studies as other researchers can conduct new analyses on the same dataset (DuBois et al., 2018). Open data sharing may also be particularly valuable for rare or small disease populations, where there is often insufficient patient data (Rubinstein et al., 2020). However, sharing data needs to be balance with confidentiality, especially with small population groups where the risk of identification is much higher (Chauvette et al., 2019).

Overall, we aimed to create and share a qualitative dataset of interview transcripts in a restricted access repository, with the aim for the data to be ‘as open as possible, as closed as necessary’ (Landi et al., 2020). In this data note, we have described the dataset to support reuse, and have reflected on the process and the limitations of our approach to open qualitative data sharing.

## Materials and methods

### Sample

We conducted semi-structured, one-to-one interviews with participants. We recruited people to the interviews from the UK, who were aged 18 or over, and either had Lynch syndrome, or were a healthcare professional involved in the Lynch syndrome care pathway. In the Lynch syndrome group, we aimed to recruit a mixture of people who took aspirin daily and those who did not. People without a diagnosis of Lynch syndrome were excluded. In the healthcare professional group, we recruited general practitioners (GPs) in primary care, community pharmacists, genetic counsellors, nurse practitioners, and specialist hospital clinicians (e.g. clinical genetics, gastroenterology). We excluded healthcare professionals deemed to be irrelevant to the Lynch syndrome care pathway, such as those who do not have contact with patients with Lynch syndrome, or those not involved in the advising or prescribing of aspirin. Overall, we recruited and interviewed 15 people with Lynch syndrome, and 23 healthcare professionals.

### Measurements

We employed semi-structured interview schedules to guide the discussions with participants, with different schedules used for the two participant groups (i.e. Lynch syndrome, healthcare professionals). The interview schedule questions were developed to cover the domains in the Theoretical Domains Framework (Atkins et al., 2017). The framework is derived from multiple behaviour change theories, and identifies the factors amenable to change that influence behaviour when implementing new clinical practices. The main aim of the interviews was to explore the barriers and facilitators towards aspirin for colorectal cancer prevention. In addition, many participants with Lynch syndrome discussed different aspects of their condition, including their family history of cancer, how they were diagnosed, and their engagement in other preventive strategies. The healthcare professional interviews also provide in-depth data on how new guidance, such as the NICE guidance on aspirin, can be implemented into clinical practice and the barriers to this.

In the healthcare professional interviews, we presented different scenarios which described a hypothetical encounter with a patient with Lynch syndrome enquiring about aspirin. We then explored healthcare professionals’ initial thoughts to these scenarios. We presented these clinical vignettes to healthcare professionals in anticipation that many would not have previous experience in this topic area. In particular, we expected the majority of GPs and community pharmacists to be unfamiliar with Lynch syndrome and using aspirin for this purpose. For example, survey studies have found that most GPs have not seen a patient with Lynch syndrome in clinic, and most are unaware that aspirin can prevent colorectal cancer in Lynch syndrome (Lloyd et al., 2023; Smith et al., 2017). In contrast, the interviews with people with Lynch syndrome instead explored participants’ experienced barriers and facilitators towards taking aspirin. The interview schedules for both groups are available in a publicly open repository (Lloyd, 2022).

Interviews were conducted with participants over video or telephone from November 2020 to November 2021. All interviews were audio-recorded using either Microsoft Teams or an encrypted Dictaphone. Interviews were transcribed verbatim by an external transcription company with a data processing agreement with the study team. The interview transcripts were stored as a Word document. The full methods of the study are reported in a previous publication with the findings (Lloyd et al., 2022).

### Consent and ethics

Ethical approval for the study was granted by the University of Leeds School of Medicine Research Ethics Committee (MREC 19-091). In the consent form, participants could provide explicit consent for their anonymised data to be deposited in the University of Leeds Restricted Access Data Repository (RADAR), with access restricted to people using the data for non-commercial research, learning, or teaching purposes. Further detail on the data sharing process was provided in the Participant Information Sheet. Participants who did not consent to this statement were still eligible to take part in the study, but their interview transcript was not uploaded to RADAR.

## Data description

Out of 15 participants with Lynch syndrome, 12 consented to their transcript being stored in RADAR. All healthcare professionals except two (one pharmacist, one GP) consented to their transcript being stored. However, we could not deposit all transcripts due to concerns on identifiability. This was because many recruited healthcare professions contained a small number of participants (e.g. one gastroenterologist, one gynaecologist). To ensure anonymity, it was decided that only the GP interviews would be uploaded to RADAR, as this was the largest healthcare professional participant group. In total, 12 interview transcripts with people with Lynch syndrome, and eight interview transcripts with GPs were uploaded to RADAR. The full demographic characteristics of the participants in the RADAR dataset are publicly available (Lloyd, 2022).

To safeguard the identity of participants, all transcripts were anonymised with names, locations, job titles, place of work, and other identifiable details removed. For example, where a name was removed in the transcript, the word [name] was inserted instead. There were heightened safeguarding concerns when anonymising the data due to interviewing people from a small population sample (Chauvette et al., 2019; Norris et al., 2024). In the Lynch syndrome interviews, participants described identifiable details regarding their family members, such as which family members have had which cancers. To anonymise these data, when a particular family member was discussed with regard to their family history of cancer, the term [Family member] was used to remove any identifiability. We also removed potentially identifiable details such as particular cancers the participant or their family members had experienced, and replaced this information with [cancer]. This was because some participants described experiencing a particular combination of different cancers that may be identifiable, or had experience with a rare type of cancer. We did not remove the term ‘colorectal cancer’ or ‘bowel cancer’ however, as this is a very common cancer among those with Lynch syndrome. The full anonymisation guide for these transcripts in available in a public open access repository (Lloyd, 2022).

Pre-registering a qualitative study before data collection commences has been described as another aspect of open qualitative data sharing (Branney et al., 2023). Before collecting data, we pre-registered our study on Open Science Framework (Lloyd & Smith, 2020), using a pre-specified template designed for qualitative pre-registration (Kern & Gleditsch, 2017). We detailed in the pre-registration factors such as the aim of the study, the target participants, how we would generate the study data, and our analysis plan. In quantitative research, the benefits of pre-registering include that it can allow other researchers to scrutinise whether questionable research practices have been employed (Nosek et al., 2018), such as HARK-ing (hypothesising after results are known) (Kerr, 1998). However, these concerns are less applicable to qualitative research which is typically hypothesis generating. Increasing the transparency of any study is of value though, including in qualitative research (Haven & Van Grootel, 2019). Pre-registering studies allows for a more in-depth discussion of the methods and analysis plan than is likely possible in the published paper, due to the strict word limits of journals.

## Discussion

To summarise, we created a qualitative dataset of interview transcripts exploring the views of healthcare professionals and people from a small genetic population. These data were deposited in a restricted access repository. There are a number of benefits to sharing research data. In quantitative research, these include verifying the data by reproducing the analysis using the data and code (McKiernan et al., 2016). While data verification typically does not translate well to qualitative research due to its subjective analytic approach (Tsai et al., 2016), there are other benefits to openly sharing data. These include retaining valuable data resources, which have often been publicly funded (McKiernan et al., 2016), and promoting data reusability to accelerate discovery in research (Landi et al., 2020). Openly sharing qualitative data from small and under-researched groups also provides the opportunity for these participants’ voices to be heard (Norris et al., 2024). This can enable other researchers to examine participants’ experiences without imposing additional burden on these groups.

While open qualitative data helps to support reusability, these data often need to be safeguarded to ensure the privacy of the subjects (Landi et al., 2020), especially in small or rare populations (Chauvette et al., 2019; Norris et al., 2024). To protect the identity of the participants, we anonymised the transcripts and deposited these in the University of Leeds restricted access repository, RADAR. The repository abides by the principles of FAIR data (Wilkinson et al., 2016). Our study data is Findable with a persistent DOI (Lloyd, 2022); Accessible to other researchers by application through RADAR; supports elements of Interoperability by publishing metadata, such as the anonymisation guide; and Reusable for non-commercial research, teaching or learning purposes. Another strength of our approach is that participants could take part in the interview without having to consent to their data being shared in RADAR. Providing participants with the option to decide which data is shared is an ethical approach recommended by the UK Data Service (2024). Furthermore, requiring consent to share data may dissuade some participants from taking part in the study, resulting in a selection bias. For example, previous research has found that people from indigenous communities can be resistant to their data being ‘open’, due to indigenous knowledge being historically misused and exploited (Albornoz & Chan, 2018). Where possible, it is essential to provide participants with the option for their data to be shared, while fully informing them on what this process entails and the benefits to open data sharing. Reassuringly, when research participants have been asked for their views on sharing their qualitative data, the vast majority supported their deidentified data being shared, and many assumed this was already a standard process (Mozersky et al., 2020).

We encountered several challenges to providing a high quality, open qualitative dataset. Similar to concerns raised by other researchers (Saunders et al., 2015), we found the process of anonymising qualitative data to be time-consuming, as the data needs to be thoroughly reviewed to ensure that all identifiable information is removed and replaced with standardised text (e.g. [place], [family member]). By fully anonymising all aspects of these data, this led to us removing important, rich data from the transcripts, which could hinder future reuse of the dataset. In addition, a number of healthcare professional interview transcripts were not deposited due to our concerns that these transcripts could be identifiable. While anonymising data is important to protect participants’ identity, over-anonymising can lead to the data being devoid of meaningful content (Tsai et al., 2016), and may prevent optimal future reuse of the data.

There are solutions to the challenge of over-anonymising qualitative data, which have been evaluated in other studies. For example, the Timescapes Archive is a specialised repository for qualitative, longitudinal data (Neale & Bishop, 2012). Due to several researchers’ concerns regarding over-anonymising qualitative data, the archive has multiple levels of access with more secure access for highly sensitive or un-anonymised data. These four levels are public access, registered access, approved access, and embargoed. In total, the archive contains longitudinal, qualitative data on over 300 individuals in the UK, all of whom consented to their data being shared. Archives such as these demonstrate the huge potential for qualitative data, at all levels of anonymisation, to be shared in some capacity. Future research could benefit from exploring the perspectives of researchers who have shared their qualitative data to explore in-depth the challenges and best practices in this area. Additionally, further research is needed to explore the impacts of openly sharing qualitative data, such as whether and how these datasets are being reused by other researchers. In turn, this will help clarify the value of openly sharing qualitative data within the research landscape.

We encourage researchers planning to share their qualitative data to carefully consider the levels of data access, and whether removing all identifiable information is necessary given their chosen data storage. Researchers should be aware that fully anonymising qualitative data may in turn limit the potential future reuse of the data. Above all, researchers should clearly communicate the level of anonymisation and data access to participants before data collection, to ensure consent is fully informed. Only with this crucial first step can qualitative data sharing begin.

## Data Availability

Public access to the interview schedules, anonymisation guide, and the participant demographic table, and restricted access to a subset of anonymised interview transcripts, are available from University of Leeds Research Data Repository: 10.5518/1097. Requests to access the transcripts in RADAR can be made at: https://radar.researchdata.leeds.ac.uk/25/. The transcripts can only be used for non-commercial research, learning, or teaching purposes, as consented by the study participants. The participant information sheet and consent form used in the study is publicly available from https://osf.io/kdq48/.

## Open Scholarship

This article has earned the Center for Open Science badges for Open Materials and Preregistered. The materials are openly accessible at https://doi.org/10.5518/1097 and https://doi.org/10.17605/OSF.IO/3EFG7.
